# Direct electrohydraulic lithotripsy with a novel peroral cholangioscope through the overtube for surgically-altered anatomy

**DOI:** 10.1055/a-2362-0579

**Published:** 2024-07-29

**Authors:** Kosuke Hiroe, Shuhei Shintani, Takuya Okamoto, Hidenori Kimura, Takaaki Eguchi, Yoshihisa Tsuji, Osamu Inatomi

**Affiliations:** 1 13051 Medicine, Shiga University of Medical Science, Otsu, Japan; 2 13051 Endoscopy, Shiga University of Medical Science, Otsu, Japan; 3 13051 General Medicine, Shiga University of Medical Science, Otsu, Japan

Endoscopic management of choledocholithiasis in patients with surgically altered anatomy is challenging and likely to be difficult 1 2 . Although the mother–baby type of peroral cholangioscopy is a useful modality, there are some limitations when using the mother scope, related to the working channel diameter and extended scope length 3 4 . In this video, we present a direct electrohydraulic lithotripsy (EHL) using a novel type of cholangioscope without a mother scope in a patient with Roux-en-Y reconstruction.

The patient was a 73-year-old woman with a history of subtotal stomach-preserving pancreaticoduodenal resection for pancreatic cancer 5 years previously. The patient had a history of endoscopic treatment of a large common bile duct stone, but complete stone extraction was difficult even after several endoscopic procedures. The stone was accompanied by recurrent liver dysfunction and obstructive jaundice. Computed tomography showed a stone with high attenuation in the hilar bile duct ( Fig. 1 ). Stenosis of the choledochojejunal anastomosis was confirmed by balloon-assisted enteroscopy, and cholangiography showed a defect of almost 20 mm in size ( Fig. 2 ). The enteroscope was removed leaving the overtube (ST-SB1S, outer diameter 13.2 mm, length 960 mm; Olympus, Tokyo, Japan) and stiff guidewire in place, and a novel cholangioscope (EyeMax, 11 Fr; Micro-Tech, Nanjing, China) ( Fig. 3 ) was directly inserted into the overtube ( Video 1 ). The novel cholangioscope combined good pushability and flexibility, which enabled easy passage through the overtube bends. EHL (Autolith Touch; Boston Scientific, Marlborough, USA) without a mother scope was initiated. The large-diameter channel (1.8 mm) simplified suction and water delivery during the procedure, with an excellent endoscopic view. It was possible to crush all the stones in one session, and we used a basket and balloon catheter to finally succeed in performing a normal complete stone extraction.

**Fig. 1:**
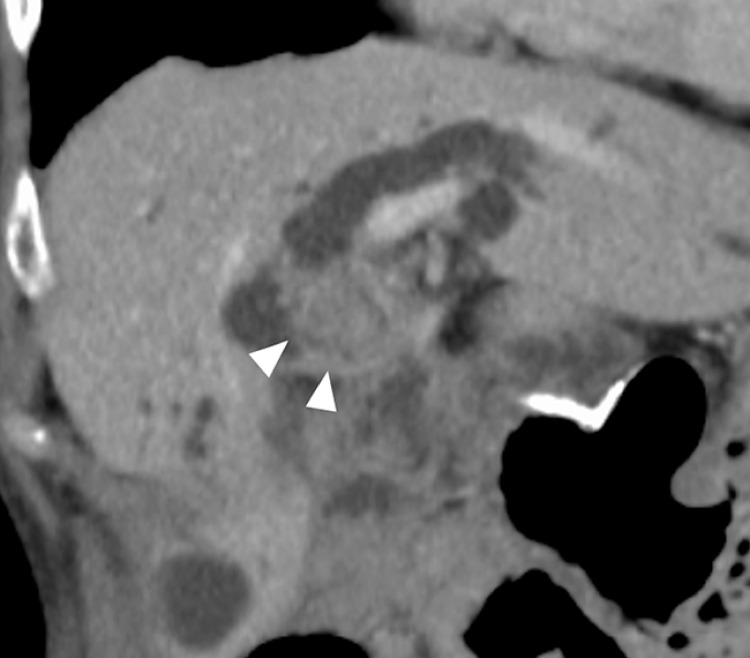
Computed tomography in a 73-year-old patient with Roux-en-Y anatomy showed a 20-mm choledocholithiasis in the hilar bile duct (white arrowheads).

**Fig. 2:**
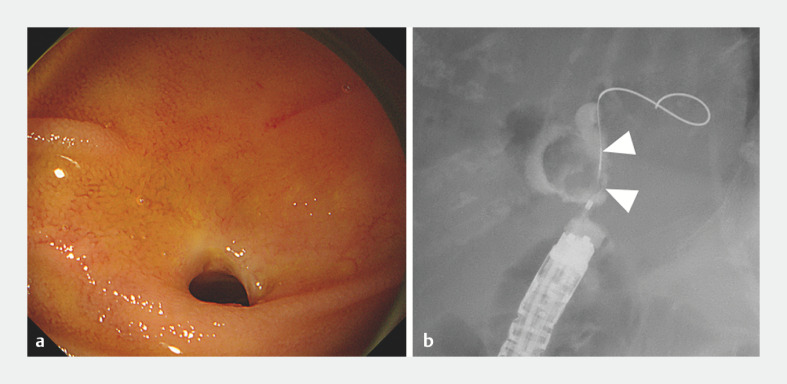
**a** Single-balloon enteroscopy-assisted endoscopic retrograde cholangiopancreatography enabled deep insertion and confirmation of a slight stricture of a choledochojejunostomy anastomosis. **b** Cholangiography showed a 20-mm defect (white arrowheads).

**Fig. 3:**
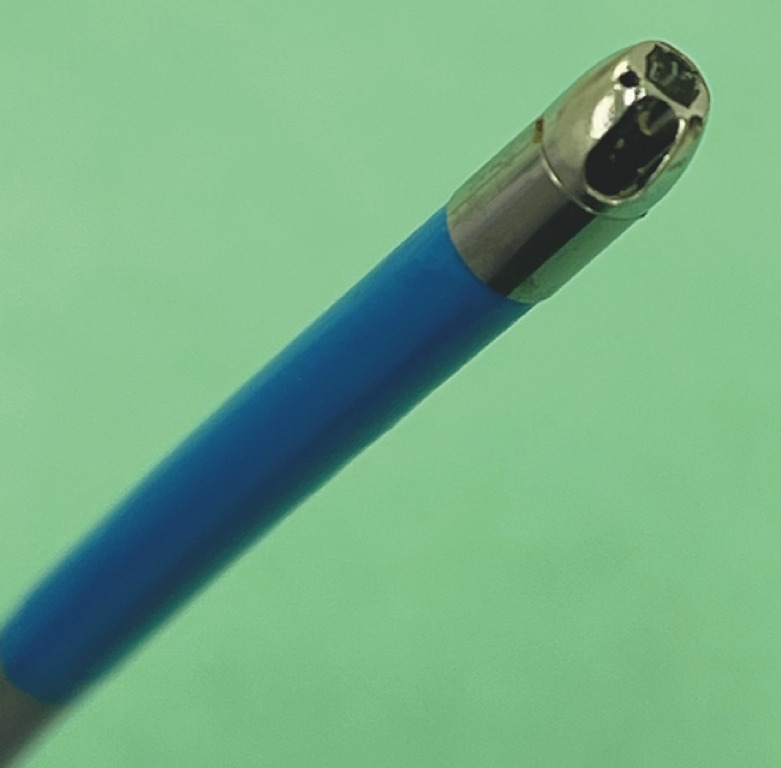
The novel direct cholangioscope has a diameter of 11 Fr and a 1.8-mm working channel (EyeMax; Micro-Tech, Nanjing, China).

Direct electrohydraulic lithotripsy (EHL) using a novel type of direct cholangioscope, without a mother scope, in a patient with surgically altered gastrointestinal anatomy.Video 1

Direct EHL with the new peroral cholangioscope proved beneficial for the management of challenging bile duct stone in a patient with altered gastrointestinal anatomy.

Endoscopy_UCTN_Code_TTT_1AR_2AH

## Notes

Endoscopy E-Videos https://eref.thieme.de/e-videos
*E-Videos* is an open access online section of the journal *Endoscopy* , reporting on interesting cases and new techniques in gastroenterological endoscopy. All papers include a high-quality video and are published with a Creative Commons CC-BY license. Endoscopy E-Videos qualify for HINARI discounts and waivers and eligibility is automatically checked during the submission process. We grant 100% waivers to articles whose corresponding authors are based in Group A countries and 50% waivers to those who are based in Group B countries as classified by Research4Life (see: https://www.research4life.org/access/eligibility/ ). This section has its own submission website at https://mc.manuscriptcentral.com/e-videos .
